# Present Situation and the Future Development of Web-Based Prenatal Education in China: Cross-sectional Web-Based Survey

**DOI:** 10.2196/28637

**Published:** 2022-06-30

**Authors:** Xinyu Huang, Weiwei Sun, Renyu Wang, Huailiang Wu, Shinning Yu, Xuanbi Fang, Yiyan Liu, Babatunde Akinwunmi, Jian Huang, Wai-kit Ming

**Affiliations:** 1 International School Jinan University Guangzhou China; 2 School of English for International Business Guangdong University of Foreign Studies Guangdong China; 3 Department of Public Health and Preventive Medicine School of Medicine, Jinan University Guangzhou China; 4 Faculty of Social Science The Chinese University of Hong Kong Hong Kong China (Hong Kong); 5 Maternal-Fetal Medicine Unit, Department of Obstetrics and Gynecology Brigham and Women’s Hospital Boston, MA United States; 6 Singapore Institute for Clinical Sciences, Agency for Science Technology and Research Singapore Singapore; 7 Department of Infectious Diseases and Public Health Jockey Club College of Veterinary Medicine and Life Sciences City University of Hong Kong Hong Kong China (Hong Kong)

**Keywords:** web-based prenatal education, pregnancy, prenatal, information technology

## Abstract

**Background:**

Pregnancy serves as an important chapter in the life of women since more attention needs to be paid to both their physical and psychological health during this period. Adequate prenatal knowledge plays a key role in ensuring the health and safety of not only the pregnant women but also their fetuses and the entire family. With the development of information technology, web-based prenatal education has been brought into focus owing to its accessibility to comprehensive information, with high-quality information available to improve the quality of the overall gestation period, labor process, perinatal outcomes, and fetal outcomes.

**Objective:**

This study aims to investigate the present situation of web-based prenatal education and to predict the future research direction of web-based prenatal education in China, thereby providing insights into improving the quality of health care of pregnant women.

**Methods:**

A national cross-sectional study was conducted on 590,912 pregnant women in 31 provincial administrations of mainland China between August 2018 and August 2019. These pregnant women were initially recruited from local hospitals across the nation during antenatal and postnatal periods via a web-based education school. Demographic information and course completion status (including the categories and the number of courses they completed) of all the participants were collected.

**Results:**

A total of 590,912 pregnant women participated in the web-based prenatal education in 2018. Among them, 188,508 (31.90%) participants were excluded because they did not complete any course, while 17,807 (3.01%) actively participated in web-based prenatal education and completed more than 100 courses. There were 5 categories of web-based courses; almost half of the pregnant women attended the courses on first and second trimesters (293,262/590,912, 49.63% and 298,168/590,912, 50.46%, respectively). We found that pregnant women were more concerned about the gestational diet, fetal-related knowledge, and other precautions before the labor.

**Conclusions:**

In the era of digitalization where information is rapidly disseminated, web-based prenatal education could become a more convenient, productive, and effective pathway for pregnant women since it could help them obtain adequate and optimal pregnancy-related information and gain more intellectual awareness about their pregnancy or preparation for pregnancy.

## Introduction

Prenatal information plays a vital role in health decision-making of pregnant women because they come across a series of physiological and psychological changes during pregnancy [1,2]. The quantity and quality of prenatal care information have been reported to be associated with the quality of life of pregnant women and the fetal outcomes in the short term and long term [3-5]. Specifically, high-quality health information is associated with better and safer pregnancy outcomes such as less preterm deliveries, less anxiety problems, lower cesarean section rates, lower maternal and infant mortality, as well as greater prenatal engagement of their partners [6]. Therefore, it is of great significance for pregnant women to gain and understand adequate and high-quality prenatal health information as much as possible.

Adequate and high-quality prenatal education is necessary for pregnant women to obtain reliable information and achieve desirable pregnancy outcomes as well as uneventful postnatal courses. However, the benefits and effectiveness of conventional prenatal education are still limited and inconclusive to some extent owing to the inadequacy in quality and quantity [7,8]. With the development of information technology, web-based prenatal education has received considerable attention. Compared to traditional group prenatal education, structural and well-organized web-based prenatal education programs can provide an easier access to medical services and integrated information, which could promote the utilization of medical sources, lower the overall educational costs, increase family engagement and satisfaction, and thereby improve the quality of daily life for pregnant women [9]. In mainland China, there were more than 900 million smart device users in 2020, and the idea of adopting web-based education for pregnancy health care–related services was increasingly viable [9]. Although there are extensive advantages, the current situation of Chinese pregnant women participating in web-based prenatal education is not known because only few studies have investigated the use of web-based prenatal education programs [10-12]. Therefore, in this study, we aim to provide insights into the present situation and future development of web-based prenatal education in China by screening the data collected from a nationwide web-based survey.

## Methods

### Recruitment

This study was designed as a nationwide cross-sectional study. Pregnant women from 31 provinces of mainland China were recruited via a web-based prenatal education school (Banmi web-based maternity school). This web-based system was allowed to assess participants’ health conditions from local hospitals and provide primary health care education to these participants through the website during their pregnancy. A total of 280 courses were offered by this web-based prenatal school, each of which took about 5 minutes to finish in average. All courses were available at any point during pregnancy. The courses transfer the information passively, but the pregnant women can take some quiz questions for self-examination after finishing each course, or they can consult with their obstetricians when they went to the hospital for antenatal examinations. The courses contained text and video, and they were free of charge to all women. All the results on course participation were based on back-end data from this web-based course system.

### Participants

The eligibility inclusion criteria included (1) pregnant women registered with the web-based prenatal education school, (2) pregnant women residing in mainland China, and (3) the expected date of delivery was between August 1, 2018 and August 31, 2019. Informed consent for research was obtained from each participant when they registered with the web-based system.

### Ethics Approval

Ethics approval was granted by the Institutional Review Board of the First Affiliated Hospital of Sun Yat-Sen University (ICE-2017-296). All procedures were conducted in accordance with the Declaration of Helsinki.

### Variables

Participants’ demographic data, including gestational age and residential location, were collected. The web-based prenatal education program categorized these prenatal courses into 5 groups: preparation for pregnancy, first trimester, second trimester, third trimester, and postpartum care. Data on course completion conditions were also collected, including the category and the number of completed courses.

### Data Analysis

We performed descriptive analyses on the data collected via the web-based prenatal education school on SPSS Statistics 25.0 for Windows (IBM Corp). We reported mean (SD) and ranges for variables that followed a normal distribution. The number of pregnant women in each province was retrieved from the China Health Statistics Yearbook 2019.

## Results

**Figure 1:**
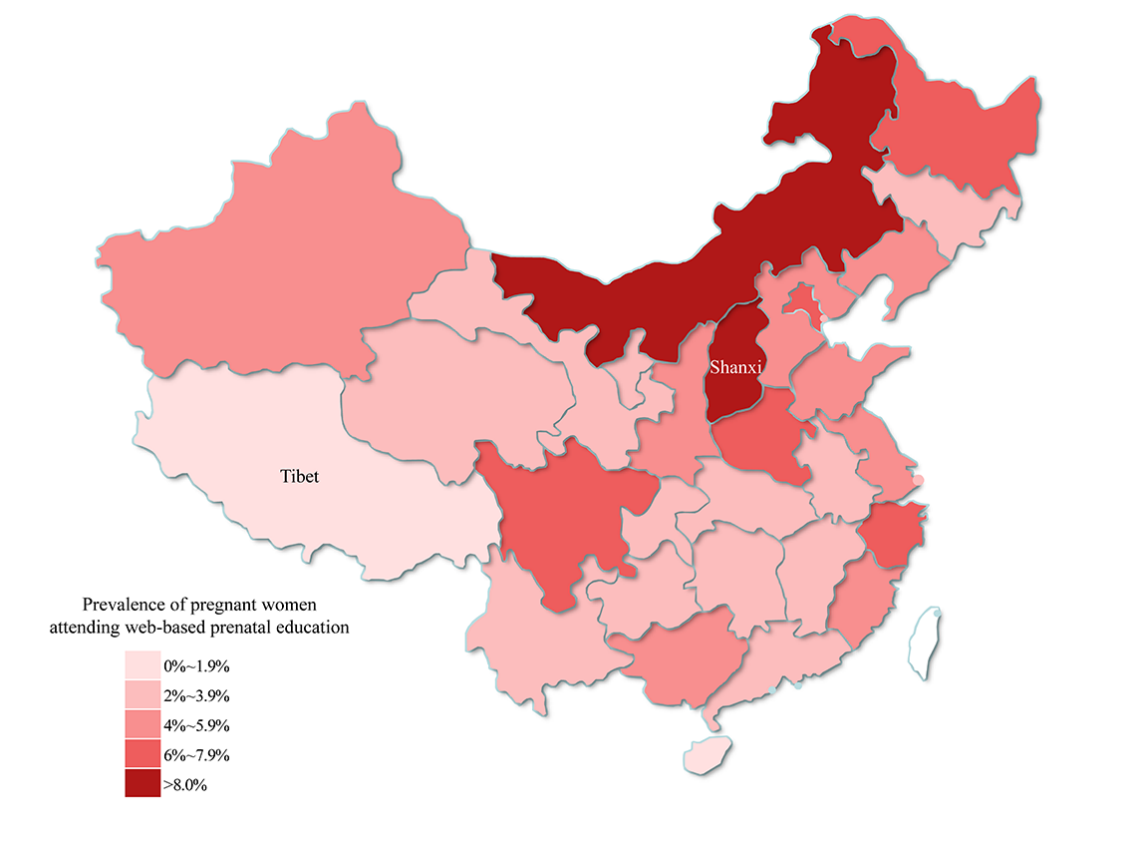
Heatmap for the prevalence of pregnant women attending web-based prenatal education in different Chinese provincial administrations.

In 2018, 590,912 women were included in this study. Figure 1 shows the heatmap of the proportion of pregnant women registered with the web-based prenatal education school in 31 Chinese provincial administrations. The region-specific proportion of pregnant women registered with the web-based prenatal education school in mainland China ranged from 0.14% (81/56,622) in Tibet to 10.46% (31,772/303,647) in Shanxi (Table 1). The proportion of pregnant women attending web-based prenatal education was less than 5% (590,912/13,621,475) in 22 (71%) of the 31 provincial administrations.

Of the 590,912 pregnant women, 188,508 (31.90%) pregnant women did not complete any course, while 136,938 (23.27%) pregnant women completed 10-100 courses, which we defined as medium participation. There were 247,659 (41.91%) pregnant women who completed only 1-10 courses in web-based prenatal education. We defined this as low participation, and that segment accounted for the largest portion of the population. Only 17,807 (3.01%) pregnant women completed more than 100 courses in the web-based prenatal education school; this was defined as high participation. Among them, 24,761 (4.19%) pregnant women participated in the course on preparation for pregnancy, 293,262 (49.63%) participated in the course on the first trimester, 298,168 (50.46%) participated in the course on the second trimester, 82,726 (14%) participated in the course on the third trimester, and 154,327 (26.12%) took the course on postpartum care.

More than half of the pregnant women attended the courses on gestational diet, fetal-related knowledge, and other precautions before the labor (Table 2). As for dietary educational courses, of the 590,912 pregnant women, 101,890 (17.24%) attended the course on “diet in second trimester” and 81,653 (13.75%) attended the course on calcium supplements, diet restrictions, anemia, and iron supplements during pregnancy. Among these pregnant women, 16.94% (100,109/590,912) were eager to obtain knowledge on fetal kicks and fetal movement counting. Approximately 13.20% (78,009/590,912) attended the courses on screening for Down syndrome, and 12.40% (73,301/590,912) were eager to understand the knowledge of screening for deformity and other congenital malformations. Besides, these pregnant women also attended the courses on behavioral change and precautions during pregnancy, such as body changes, sexual behavior during pregnancy, as well as contraindications and cautions on harmful things for pregnant women.

Apart from the courses with the highest number of participants, we also calculated the 10 courses with the least participants, as shown in Table 3.

From this table, we can observe that the courses related to pregnancy diseases were attended by less number of pregnant women. Less than 1% of the pregnant women attended the courses on gestational diabetes, premature delivery, female infertility, extrauterine pregnancy, and polycystic ovarian syndrome.

**Table 1:** Proportions of pregnant women completing web-based prenatal education in mainland China in 2018.a

Provincial administration			Pregnant women (N)^a^		Pregnant women attending web-based prenatal education, n (%)
**North China**					
	Shanxi	303,647		31,772 (10.46)	
	Inner Mongolia	181,445		15,311 (8.44)	
	Beijing	140,304		10,668 (7.60)	
	Hebei	735,253		34,914 (4.75)	
	Tianjin	78,072		2765 (3.54)	
**Northeast China**					
	Heilongjiang	151,058		9828 (6.51)	
	Liaoning	253,180		13,405 (5.29)	
	Jilin	136,396		3399 (2.49)	
**East China**					
	Zhejiang	413,967		27,061 (6.54)	
	Fujian	444,863		21,272 (4.78)	
	Jiangsu	619,047		26,037 (4.21)	
	Shandong	1,058,022		42,893 (4.05)	
	Shanghai	69,734		2505 (3.59)	
	Jiangxi	558,666		19,513 (3.49)	
	Anhui	736,530		16,805 (2.28)	
**Middle China**					
	Henan	1,126,750		65,987 (5.86)	
	Hubei	590,923		21,321 (3.61)	
	Hunan	705,524		18,828 (2.67)	
**South China**					
	Guangxi	667,539		36,975 (5.54)	
	Guangdong	1,317,909		38,879 (2.95)	
	Hainan	101,307		1076 (1.06)	
**Southwest China**					
	Sichuan	800,752		50,241 (6.27)	
	Yunnan	564,411		16,060 (2.85)	
	Chongqing	273,940		7689 (2.81)	
	Qinghai	74,287		1689 (2.27)	
	Tibet	56,622		81 (0.14)	
**Northwest China**					
	Shaanxi	346,083		19,634 (5.67)	
	Xinjiang	197,954		8497 (4.29)	
	Gansu	278,912		10,102 (3.62)	
	Ningxia	73,409		2524 (3.44)	
	Guizhou	564,969		13,180 (2.33)	
Overall			13,621,475		590,912 (4.34)

^a^Source: China Health Statistics Yearbook 2019.

**Table 2:** Top 10 popular courses among pregnant women (N=590,912).

Course	Participants, n (%)
Diet in the second trimester	101,890 (17.24)
Fetal movement counting	100,109 (16.94)
Body change in the second trimester	99,895 (16.91)
Calcium supplement	81,653 (13.82)
Diet restrictions during pregnancy	81,259 (13.75)
Sexual behavior during pregnancy	81,143 (13.73)
Anemia and iron supplements during pregnancy	78,141 (13.22)
Screening for Down syndrome	78,009 (13.20)
Screening for deformity	73,301 (12.40)
Harmful things for pregnant women	73,179 (12.38)

**Table 3:** Ten least popular courses among pregnant women (N=590,912).

Course	Participants, n (%)
How to select and use diapers	199 (0.03)
Prevention and prognosis of gestational diabetes	1779 (0.30)
How to prevent premature delivery	1841 (0.31)
Labor sign: amenorrhea	2474 (0.42)
The best age for pregnancy	2583 (0.44)
What is Doula delivery	2661 (0.45)
Pet raise during pregnancy	3149 (0.54)
Female infertility	3174 (0.54)
Reasons for extrauterine pregnancy	3225 (0.55)
Polycystic ovarian syndrome and pregnancy	3757 (0.64)

## Discussion

### Principal Findings

In this study, we found that the overall proportion of pregnant women registered with the web-based prenatal education school was 4.34% (590,912/13,612,475) in mainland China, while there were substantial differences from region to region. Although about one-third of the participants did not complete any course in the system, each pregnant woman completed an average of about 20 courses. In terms of course preference, half of the pregnant women were enrolled in courses on the first trimester and second trimester of pregnancy. Specifically, dietary supplements, fetal-related knowledge, and predelivery considerations such as how to protect themselves and how to schedule prenatal visits were the most popular topics.

A previous study reported that pregnant women who participated in web-based prenatal education performed better than those who did not, in many aspects during pregnancy, including daily activities and perinatal care [13]. With the prevalence and evolution of the internet and mobile devices, the amount of information available online has increased dramatically. Web-based prenatal education is favored by more pregnant women because of its convenience, accessibility, flexibility, and cost-effectiveness [14]. According to a Chinese study, pregnant women preferred evidence-based information, expert opinions, and tailored advice from the internet [15]. However, in 2018, only 4.34% (590,912/13,612,475) of the pregnant women registered with the web-based prenatal education school in our study. Such results are partly attributed to some unstandardized and unreliable scientific information on websites. In addition, people lack trust in the information available on the internet and fear being misled or misinformed [16]. Findings from a Korean study showed that 39% of internet health information was inaccurate and 42.7% of those misleading messages were irrelevant to health problems and could cause a series of negative impacts [17]. Thus, pregnant women might worry about the adverse effects of following wrong prenatal guidance from the internet [18].

Standard and certified web-based prenatal education contains a wealth of contents that have been professionally screened. Provision of accurate and appropriate prenatal knowledge to pregnant women can improve their self-care ability, and to some extent, improve their safety and physical conditions during their pregnancy [4]. On the contrary, inaccurate information from the internet can mislead pregnant women to perform behaviors that are not conducive to their health and safety. Therefore, web-based prenatal education should be evaluated and improved by the health care system, medical institutes, and professional experts, as this is a promising pathway to provide higher-quality content for pregnant women and ensuring the accuracy and quality of the web-based prenatal education programs. This will help in achieving the desired aim of protecting pregnant women, thereby improving their quality of life during pregnancy and contributing to better pregnancy outcomes. This will also help in mitigating the anxiety and fears perceived by pregnant women toward inaccurate information and therefore promote the popularization and utilization of the web-based prenatal educational services [3,5,18].

Different use levels of web-based prenatal school in different regions were also found in this study. Internet access and use is closely associated with regional economic and education levels; the educational level and internet use are relatively low in the western provincial regions in mainland China [19,20]. This may well explain our observation that pregnant women in Tibet have the lowest proportion of participation in web-based prenatal education. Furthermore, according to the China Health Statistics Yearbook 2019, the rates of record establishment, prenatal examination, and systemic management of pregnant women in Tibet were the lowest among all provincial administrations in 2018 [21]. These were associated with the poor awareness of importance of prenatal education and examinations and the lack of adequate medical and educational resources for pregnant women in Tibet [22,23]. In contrast, northern and northeastern China were the regions with the highest numbers for these figures. Meanwhile, the gap was deepened by the relatively high rate of pregnant women establishing pregnancy records and prenatal visits in these 2 regions [21].

Only 3.01% (17,807/590,912) of the pregnant women completed more than 100 courses in this study, while about one-third of the pregnant women who had registered for the web-based prenatal school did not complete any course. This indicated a suboptimal level for the completion of participation in the web-based prenatal education. This could be explained by their indifferent attitudes toward web-based prenatal education and insufficient attraction of pregnancy courses. Thus, medical institutions and hospitals should collaborate to improve the contents and services of prenatal education programs. Moreover, courses on the first and second trimesters of pregnancy are more popular among pregnant women owing to the great physical and physiological changes in these phases of pregnancy [1,24,25]. Previous studies have shown that pregnant women normally preferred searching for information online at the beginning of pregnancy [26,27], and pregnant women usually start antenatal care during the first trimester, preferably before the 12th week of gestation [22]. A study found that more than half of the Chinese pregnant women completed their first antenatal care after the first trimester [28]. To sum up the above, these may explain the preference of knowledge on both first and second trimesters, since the pregnant women in our study were generally recruited at their visit to the hospitals for antenatal care. Nevertheless, 4.19% (24,761/590,912) of the pregnant women completed the courses on the preparation for pregnancy, which indicated that some pregnant women were also concerned about the knowledge on the anticipation and preparedness of pregnancy. Therefore, obstetric experts in web-based prenatal education schools should provide web-based lectures and consultation services for women of childbearing age; consequently, there will be an expected increase in safe conception and reduction in pregnancy complications when women of childbearing age seek for help in the web-based prenatal education school prior to pregnancy, with the education and guidance from obstetric experts.

Regarding the courses completed by the participants, the top 10 popular courses mainly focused on gestational diet, fetal-related knowledge, and precautions during pregnancy. Previous studies found that fetal development and nutrition knowledge were often a concern for pregnant women, and our findings were consistent with those reported previously [26,29]. Besides, better quality of maternal diet during pregnancy is positively associated with general maternal health, reproductive outcomes, and child neurodevelopment [24,30]. Furthermore, strong correlations of incidence of gestational diabetes mellitus and type 2 diabetes and energy intake during pregnancy were widely reported [25,31,32]. These pieces of evidence suggest that medical institutes should pay more attention to dietary habit education during pregnancy to improve the quality of life during pregnancy. Besides, fetal-related courses such as fetal movement counting, screening for Down syndrome, and screening for deformity were also popular. Prenatal screening mainly focused on Down syndrome and fetal anomaly scans in the routine second trimester [33]. Thus, it is necessary for hospitals and other medical institutions to provide more education and notices on prenatal screening. At the same time, the courses related to pregnancy diseases were not attractive to pregnant women, and only less than 1% of them attended the courses on gestational diabetes, premature delivery, female infertility, extrauterine pregnancy, and polycystic ovarian syndrome. This condition may be caused by the indifferent attitude toward pregnancy-related diseases, and many of them did not have a positive cognitive attitude toward those diseases. Therefore, obstetrics physicians should inform pregnant women of common pregnancy-related diseases and how to manage them properly in a timely manner, which is also highly relevant to pregnancy health.

With the development of modern information technology, web-based prenatal education can be a good choice for pregnant women to obtain gestational knowledge of qualified quality and adequate quantity, including how to prepare for pregnancy, what should be done during the gestational period, how to effectively recover from parturition, and what challenges they will meet during the postpartum period. The number of internet users in China is nearly 1000 million, of which rural internet users is 309 million. The nationwide network coverage rate reached 70.4% by December 2020, and the network coverage rate in rural areas was 55.9%, while previous data suggested that there is still about 64.5% of Chinese pregnant women who did not attend any prenatal education [34]. The web-based health program is cost-effective to provide information, resources, and education, which could help to fill in the gap in the field of prenatal education [12,35]. For pregnant women in rural areas, web-based prenatal education can help to reduce the financial burden such as the cost of transportation and medical consultation in hospitals, providing them a more cost-effective way to improve their safety and life quality during pregnancy. Moreover, web-based prenatal education can avoid some unnecessary visits and reduce the risks of cross-infection in hospitals [36,37]. In general, web-based prenatal education provides comprehensive information to enable pregnant women to learn more about pregnancy, equip them with better self-care ability, and protect their own health and safety when facing some physiological and psychological changes during pregnancy. In addition, owing to its convenience and low cost, high-quality medical resources can be availed in any corner of the planet covered with the internet network. Web-based prenatal education provides a certain guarantee of health care for pregnant women both subjectively and objectively.

Web-based prenatal education programs could be improved in the following areas for offering a higher quality of health care for pregnant women [38,39]. First, women of childbearing age need access to log in before pregnancy—the time when they are more likely to search gestational information and gain some preconception knowledge for preparation. Second, the contents of web-based courses, especially knowledge of early pregnancy should be supplemented, as they are necessary for pregnant women to have a better quality of pregnancy. Moreover, obstetricians’ involvement and consultation could be considered as a combination strategy with web-based prenatal programs in the future. Third, governments need to build better internet facilities and provide mobile devices with greater discounts in rural areas or for underprivileged people, because these can provide essential requirements for pregnant women to experience and benefit from web-based prenatal education programs.

### Limitations

To the best of our knowledge, this is the first study to investigate current situations and provide insights into the development of web-based prenatal education programs in China. However, our study has few limitations. First, this study only included 1 national web-based prenatal school. There are about 10 local web-based prenatal schools in China, but they are mainly used in local hospitals by their own apps or official account. The Banmi web-based prenatal school mentioned in this paper is the largest one and can be accessed by everyone in mainland China. The data from this school as a reference to calculate the proportion of usage of web-based prenatal education for pregnant women might be slightly underestimated, but this result will be more accurate with the development of information technology and popularization of network. Second, all the results of course participation were based on back-end data from the web-based course system; therefore, we did not include any demographics or other maternal characteristics such as age, education, and employment status. When comparing the regional difference of proportions of using web-based prenatal education programs, this study did not account for the aforementioned factors, and these demographic factors might have great influences on the usage of web-based prenatal education programs. Besides, we did not know whether the course participation of pregnant women is driven by women’s interests or obstetric providers’ recommendations, which may influence the selection of pregnant women.

### Comparison With Prior Work

We searched PubMed, Google Scholar, and China National Knowledge Infrastructure for papers about the current development and applications of web-based antenatal care in China. Of the studies we identified, we found that web-based antenatal care currently has a variety of researches and applications in the clinic, such as psychological interventions for depression, childbirth, and breastfeeding education. However, the present situation of Chinese pregnant women participating in web-based prenatal education is not known, and large-scale population-based studies in China focused on the participation rate of pregnant women enrolled in web-based antenatal care are absent. Thus, this is a pioneer national-based study to investigate the participation of pregnant women in web-based prenatal care in China. This study emphasizes on exploring the concerns and preferences of pregnant women, providing evidence and references for future web-based prenatal education development, which can contribute to the improvement of maternal and fetal health, quality of health care for pregnant women, and overall pregnancy outcomes. In this study, we observed the participation, concerns, and preference of pregnant women when they attend web-based prenatal courses. Our study shows that web-based prenatal education has great potential in future development and application, and web-based prenatal education could become a convenient and effective pathway for pregnant women to obtain important and optimal pregnancy-related knowledge and gain more information about the pregnancy while pregnant or preparing to be pregnant.

### Conclusions

With the development and popularization of information technology, web-based prenatal education shows promising potential in future development and application. In mainland China, the usage of web-based prenatal education for pregnant women was not very high but increased rapidly. Support from the government and health care professionals can assist in the development of standard and high-quality web-based prenatal programs, which can contribute to the improvement of maternal and fetal health, quality of health care for pregnant women, and the overall pregnancy outcomes.
